# Can the Understory Affect the Hymenoptera Parasitoids in a Eucalyptus Plantation?

**DOI:** 10.1371/journal.pone.0151165

**Published:** 2016-03-08

**Authors:** Onice Teresinha Dall’Oglio, Rafael Coelho Ribeiro, Francisco de Souza Ramalho, Flávio Lemes Fernandes, Carlos Frederico Wilcken, Sebastião Lourenço de Assis Júnior, Rosa Angélica Plata Rueda, José Eduardo Serrão, José Cola Zanuncio

**Affiliations:** 1 Instituto de Ciências Agrárias e Ambientais, Campus Universitário do Tocantins (CUNTINS), Universidade Federal de Mato Grosso, Sinop, MT, Brazil; 2 Faculdade de Agronomia, Universidade Federal do Pará, Cametá, PA, Brazil; 3 Unidade de Controle Biológico, Embrapa Algodão, Campina Grande, PB, Brazil; 4 Campus de Rio Paranaíba, Universidade Federal de Viçosa, Rio Paranaíba, MG, Brazil; 5 Departamento de Proteção Vegetal, Faculdade de Ciências Agronômicas (UNESP), 18610–307, Botucatu, SP, Brazil; 6 Departamento de Engenharia Florestal, Universidade Federal dos Vales do Jequitinhonha e Mucuri, 39.100–000, Diamantina, MG, Brazil; 7 Departamento de Fitotecnia, Universidade Federal de Viçosa, 36570–900, Viçosa, MG, Brazil; 8 Departamento de Biologia Geral, Universidade Federal de Viçosa, 36570–900, Viçosa, MG, Brazil; 9 Departamento de Entomologia/BIOAGRO, Universidade Federal de Viçosa, 36570–900, Viçosa, MG, Brazil; Natural Resources Canada, CANADA

## Abstract

The understory in forest plantations can increase richness and diversity of natural enemies due to greater plant species richness. The objective of this study was to test the hypothesis that the presence of the understory and climatic season in the region (wet or dry) can increase the richness and abundance of Hymenoptera parasitoids in *Eucalyptus* plantations, in the municipality of Belo Oriente, Minas Gerais State, Brazil. In each eucalyptus cultivation (five areas of cultivation) ten Malaise traps were installed, five with the understory and five without it. A total of 9,639 individuals from 30 families of the Hymenoptera parasitoids were collected, with Mymaridae, Scelionidae, Encyrtidae and Braconidae being the most collected ones with 4,934, 1,212, 619 and 612 individuals, respectively. The eucalyptus stands with and without the understory showed percentage of individuals 45.65% and 54.35% collected, respectively. The understory did not represent a positive effect on the overall abundance of the individuals Hymenoptera in the *E*. *grandis* stands, but rather exerted a positive effect on the specific families of the parasitoids of this order.

## Introduction

Monocultures have a lower biodiversity, which may make it necessary to control insect pests with pesticides. The use of synthetic pesticides leads to accumulation of potentially toxic substances in soil, water, and food resources. The negative impacts of these compounds include reduction in the biodiversity and degradation of the ecosystem quality, which has necessitated searching for environmentally friendly and cheaper methods of pest control [1].

The landscape affects the biological control due to the migration of the predators and parasitoids from the adjacent natural habitats to the farmland [2,3]. These habitats provide resources such as alternative hosts or prey, water, food, favorable microclimate and shelter beneficial for the arthropods [4]. Food availability is an important factor to supply the energy for locomotion or flight and to maintain the longevity and fecundity of the natural enemies [5]. Herbs and shrubs [6,7], including plants such as Apiaceae, Asteraceae and Fabaceae may harbor hosts or prey [7] which can increase the diversity and abundance of the natural enemies [8]. In addition, plants, including the cultivated species have special structures as hair, domatia and nectaries that provide shelter for the natural enemies [5].

The presence of the understory vegetation in the temperate deciduous forests increased the animal and plant diversity [9]. However, the understory revealed a homogenizing effect on the composition of the fauna in the temperate forest, unlike that observed in a Chinese subtropical forest [10]. The understory can alter the foraging chances and reduce the negative interactions between the pests and natural enemies [11]. Forest ecosystems can provide organic compounds [12], prey [13], competitors [14] and predators [15] which can affect the survival of the organisms [16]. Because of these reasons, we expect the presence of the understory will increase the richness and abundance of Hymenoptera parasitoids in eucalyptus plantations which may facilitate biocontrol of pest organisms

Parasitoids reduce number of pest rather than controlling [2,3]. Therefore, the maintenance of the understory and preservation of the remnant of the native vegetation in the eucalyptus plantations have been promoted to increase the invertebrate communities with the potential for biological control [17,18]. Parasitism is normally higher in the diverse landscapes due to the greater number of nectar sources and microclimates [19,20].

Heavy rains and sudden changes between the rainy and dry season may have a negative impact on social wasp populations, caused by the lower resistance of parasitoid wasps to the associated pathogens mediated by unfavorable conditions [21]. In the dry season, the parasitism level may be reduced due to some species of Lepidoptera develop before their parasitoid associate, facilitating lepidopteran pests proliferation [22].

The objective of this study was to test the hypothesis that the presence of the understory and climatic season in the region (wet or dry) favors the richness and abundance of Hymenoptera parasitoids in *Eucalyptus* plantations.

## Materials and Methods

Sampling was conducted in ten areas of *Eucalyptus grandis* Hill ex Maiden stands with five areas with understory and five where it was absent, with 5-year-old trees in Belo Oriente, Minas Gerais State, Brazil. The spacing between plants was 3 x 2 meters, and eucalypt plantations with and without understory was surrounded by remnant of native vegetation, represented by fragments of the Atlantic Forest.

Collecting pots with 80% alcohol were placed in the traps and removed and replaced every 15 days during the drier months (March-June) and raining months (June to October). These pots were taken to the Laboratory of Biological Control (LCBI) of the Institute for Applied Biotechnology to Agriculture (BIOAGRO) of the Universidade Federal de Viçosa (UFV) in Viçosa, Minas Gerais State, Brazil.

The climate of the Belo Oriente region, Minas Gerais State, Brazil is of tropical type (Aw according to the Köppen classification), with annual average temperatures between 22 and 27°C, relative humidity of 67%, average annual rainfall from 701 to 1500 mm and an average altitude of 240 m. The eucalypt plantation had 815.50 ha, distant about 10 m by native vegetation with 1,363.83 ha, represented by fragments of the Atlantic Forest. The native understory in the area consisted of herbs of the Asteraceae, Bignoniaceae, Chrysobalanaceae, Euphorbiaceae, Heliconiaceae, Lauraceae, Leguminosae, Malvaceae, Moraceae, Poaceae, Rubiaceae, Rutaceae and Sapindaceae families. This vegetation was removed in the areas without the understory and left in those with understory. The samples were taken with Malaise traps set at 2 m height [23].

The methods, species collected and areas where the e collections used in the manuscript followed recommendations of the Instituto Brasileiro de Meio-Ambiente (IBAMA), with number of collection license SISBIO—21060–2. The material collected was sorted and quantified and the parasitoids were stored in alcohol. Those belonging to the order Hymenoptera were identified until the family level [24,25,26] and deposited in the entomological collection of LCBI/UFV.

The data on the most abundant Hymenoptera families found in the eucalyptus stands with or without the understory were fit with linear models and after confirming normal distribution of the residuals with the Shapiro-Wilk test and homogeneity of variances with the Bartlett test analysis of variances were performed. Later *t test* was performed to comparing the sites with and without understory.

## Results

**Fig 1:**
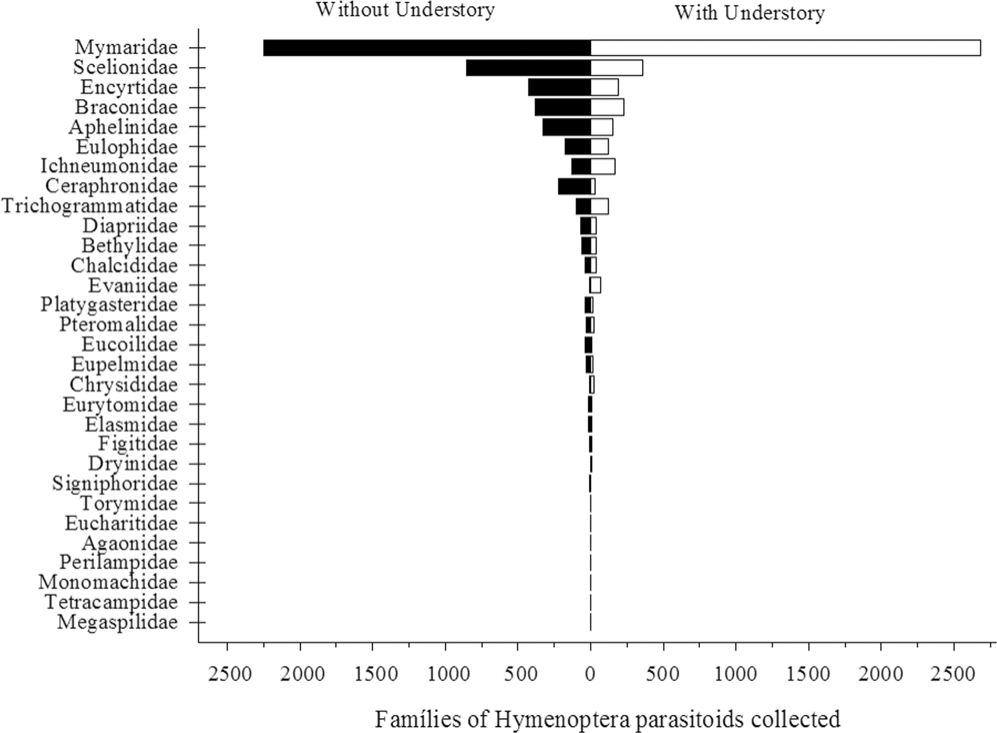
Abundance of of Hymenoptera parasitoid individuals collected with Malaise traps in *Eucalyptus grandis* stands without and with understory and total number of individuals collected, Municipality of Belo Oriente, Minas Gerais, Brazil.

A total of 9,639 individuals from 30 Hymenoptera parasitoid families were collected. Mymaridae, Scelionidae, Encyrtidae and Braconidae were the most abundant ones with 4,934, 1,212, 619 and 612 individuals, respectively (Fig 1). The relative abundance of Hymenoptera compared to all individuals collected was similar between eucalyptus stands with or without the understory, 45.65% and 54.35%, respectively. However, we did not find a general change in abundance with or without understory, but rather that some families increased while others decrease with understory.

The family Mymaridae and the superfamily Ichneumonoidea, especially Braconidae and Ichneumonidae were the most abundant in the eucalypt stands, both with or without the understory. However, the Aphelinidae, Ceraphronidae, Encyrtidae, Scelionidae families had high abundance in the eucalypt stands without an understory (Fig 1; Tables 1 and 2).

**Table 1:** Analysis of variance for the number of individuals of the high abundance families of Hymenoptera parasitoids (Families) collected in *Eucalyptus grandis* with and without understory. Municipality of Belo Oriente, Minas Gerais, Brazil.

HymenopteraFamilies	Understory		Date		Understory Vs. Date	
	F	P	F	P	F	P
Hymenoptera total	2.14	0.15	2.07	0.02*	1.14	0.33
Mymaridae	0.65	0.42	6.41	0.00*	1.46	0.15
Braconidae	6.07	0.02*	0.65	0.79	1.47	0.15
Ichneumonidae	1.06	0.30	3.03	0.00*	1.45	0.15
Scelionidae	13.86	0.00*	0.98	0.47	0.55	0.88
Encyrtidae	4.27	0.04*	1.95	0.04*	0.96	0.49
Aphelinidae	5.48	0.02*	1.45	0.15	0.84	0.60

*Values of probabilities were significant at the *0*.*05*.

**Table 2:** Mean number of individuals collected with Malaise traps type in eucalypt plantations with and without understory in Belo Oriente, Minas Gerais, Brazil.

Hymenoptera Families	With Understory	Without Understory
	Mean	
Hymenoptera total	125,97*	145,47
Mymaridae	536,60	450,20
Braconidae	46,20*	76,20
Ichneumonidae	33,00	25,20
Scelionidae	71,20*	171,20
Encyrtidae	38,60*	85,20
Aphelinidae	30,20*	64,80

*Significant with *t* test.

**Fig 2:**
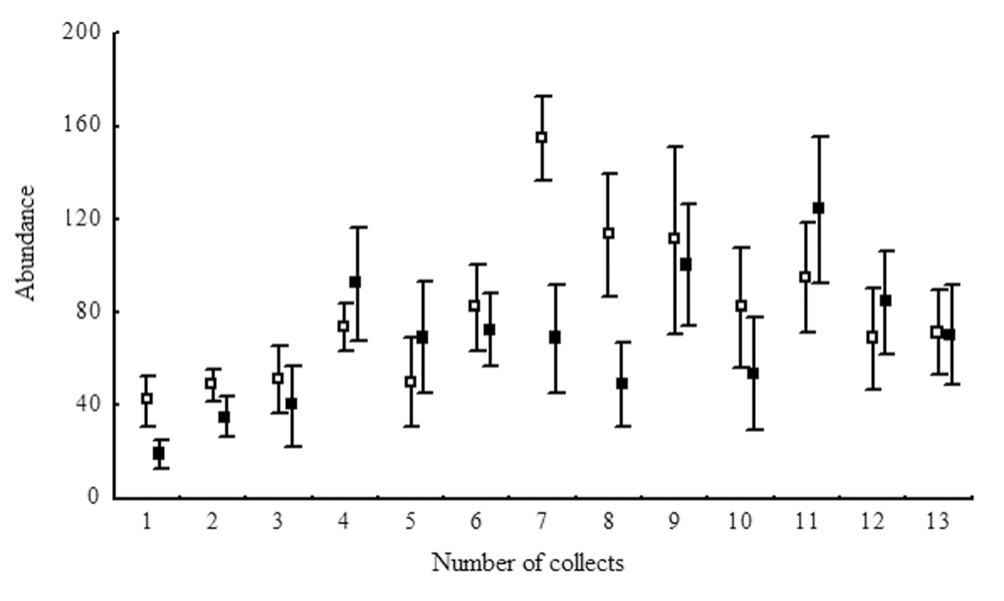
Abundance of individuals from families of Hymenoptera parasitoids collected with Malaise traps in stands of *Eucalyptus grandis* with and without understory. Municipality of Belo Oriente, Minas Gerais, Brazil. *Eucalyptus* understory—full signal, no eucalyptus understory—open signs.

The date of the collection affected the abundance of some of the Hymenoptera (Fig 2; Table 1). The family Mymaridae showed higher abundance from the fourth to the tenth collections (June-August) during the winter months in the region (Table 1). The Scelionidae, Braconidae and Aphelinidae were not affected by the sampling period (Table 1); Ceraphronidae was the most abundantfrom mid-August to the first of September; Encyrtidae had greater abundance in the eucalyptus without an understory in July (Table 1); Eulophidae had similar numbers of individuals in the eucalyptus stands with and without an understory and Ichneumonidae was had the highest abundance from August (10th. collection), but with similar numbers in the eucalyptus stands with and without the understory (Fig 2; Tables 1 and 2).

The diversity index of Berger-Parker H' for parasitoid families was higher in areas of eucalyptus without (H' = 2.01) than in those with (H' = 1.67) understory (Table 3). However, the dominance index was higher in areas of eucalyptus with understory (H' = 0.61) than among those without understory (H' = 0.43) (Table 3).

**Table 3:** Number of individuals, richness, diversity and dominance of Hymenoptera families, collected with Malaise traps type in Belo Oriente, Minas Gerais, Brazil eucalypt plantations.

	Eucalypt without Understory	Eucalypt with Understory
Number of individuals	5,239	4,400
Richness	31	31
Shannon-Wiener *H'*	2.0060	1.6630
Berger-Parker	0.4297	0.6098

## Discussion

The analysis of the family taxon of insects is important, particularly in the forest environments for conservation, planning and biomonitoring [27,28]. Hymenoptera family level becomes important because there are many with parasitoids species with unique habit, as Braconidae, Chalcididae, Encyrtidae, Ichneumonidae, Mymaridae, Scelionidae and Trichogrammatidae, which may favor natural biological control in cultivated eucalyptus [29,30,31,32,33]. The large number of Hymenoptera families with individuals captured in the *E*. *grandis* plantations, with or without an understory, indicates the presence and diversity of the lepidopteran defoliators or other arthropods groups in these forest systems [34,35], because of their association with their hosts [36]. The physical factors of the habitat, cultural practices and pesticides to control the pests and the diseases can affect the diversity of the parasitoids [37]. In addition, plants can provide essential resources and shelter to these organisms, which can affect their abundance and survival, attack rate to herbivores and parasitoid fecundity, crucial for the efficiency and frequency of these biological control agents [5].

The similarity in the abundance of the Hymenoptera parasitoids in the eucalyptus stands with or without an understory may reflect the fact that their populations are originated from the fragments of the native forest [38] surrounding the plantations. This can increase the biodiversity in the crop area acting as the reservoirs and biological corridors of movement and distribution of the natural enemies in the eucalyptus plantations [7,39,40]. Thus, fragments of the native vegetation between the eucalypt stands could maintain the populations of the natural enemies even when their hosts become scarce in the eucalyptus plantations, besides allowing their migration to the interior of such plantations [41]. The fusion of the conservation areas can facilitate the dispersal of the insects to the eucalyptus stands, especially those of the natural enemies [42]. A mosaic landscape composed of both cultivated and fragments of native vegetation can create multiple habitats for the breeding, feeding and sheltering of the natural enemies [7,43]. This can improve the environmental stability in the homogeneous stands [18], and reduce the lepidopteran defoliator populations in the forest plantations [44]. Areas of the native vegetation between the forest plantations is a technique of the integrated management of insect pests in the eucalyptus plantations in Brazil [45], in which the study area included about 46.87% of its surface with preserved native forest among the eucalyptus stands.

The greater abundance of the Aphelinidae, Ceraphronidae, Scelionidae and Encyrtidae in the areas of *E*. *grandis* without an understory may be due to the host stages preferred by natural enemies of these families. The egg, larva and pupa stages are more exposed and their movements are easily identified in these environments [7, 46]. The shortness in height (2 m) of flight interception traps (Malaise) used in the research, trapped mainly the smaller Hymenoptera as Braconidae, Encyrtidae, Mymaridae and Scelionidae, similar to the results of the research conducted with bottle traps at the ground level [47]. This can be explained by the fact that these Hymenoptera are not very mobile, being positive phototropic and geotropic negatives. Such traits are likely to be crucial for the collection of individuals of these families. [48]. However, this differs from those with the traps at least 5 m in height to collect insects in the canopy which are more viable for collecting the larger species, such as individuals of the Vespidae family [49].

The highest dominance index (Berger-Parker H') of Hymenoptera parasitoids in areas with understory was due to that parasitoids, especially to those of Mymaridae family, are better adapted to exploiting features as nectar and pollen [50] and refugees in understory plants during the culture disturbance [51,52].This confirms that more complex environments have typically a larger number of niches and resources [53,54]. The diverse vegetation of the understory can facilitate the development of natural enemies that can maintain high biocontrol rates in forest plantations [55,56,57]. The presence of fragments of native forest surrounding the *E*. *grandis* plantation favored quality in both systems evaluated (absence and presence of understory). However, the greatest diversity index (Shannon-Wiener H') of Hymenoptera parasitoids families in areas without understory can be explained by the lack of vegetation barriers such as herbs and shrubs that comprise the understory. This could have allowed the parasitoid flight to areas without understory and therefore favoring high Hymenoptera family diversity [58].

The higher frequency of the Mymaridae family, especially found in the *E*. *grandis* stands without an understory, concurs with that reported for these parasitoids in the understory, edge and interior of a tropical forest in Central Amazonian [59] and in the understory of the temperate forests in northeastern Canada [48]. The Mymaridae are exclusively egg parasitoids of Coleoptera, Diptera, Hemiptera, Lepidoptera, Odonata, Orthoptera, Psocoptera and Thysanoptera, including the bronze-bug, *Thaumastocoris peregrinus* Carpintero & Dellape (Hemiptera: Thaumastocoridae) a major insect pest of the eucalyptus plantations in Brazil [60]. Although poorly known, due to its small size, the species of this family are a very common and diverse group [61].

The abundance of Scelionidae in the eucalyptus stands, especially without an understory is noteworthy because this family includes the egg parasitoids of agricultural and forest pest species [62]. *Trissolcus basalis* (Wollaston) and *Telenomus podisi* Ashmead (Hymenoptera: Scelionidae) are the most important egg parasitoids of the phytophagous [63] and predatory (Heteroptera: Pentatomidae) bugs [64].

Encyrtidae species, which were abundant in the eucalyptus stands without an understory, also had high abundance in temperate forests and in the Island of Borneo [48]. This family includes important species for the biological control of pests, especially those of Coleoptera, Diptera, Hemiptera and Lepidoptera [65].

The low variation in the number of individuals of some parasitoid families of Hymenoptera as Bethylidae, Chalcidae, Figitidade, Pteromalidae, etc. over time, in the eucalyptus stands with an understory shows their greater stability in this plantation system, which may have been aided by the lower disturbance in the ecosystem that boasted no cultural practices such as cleaning the area [66].

The high number of the individuals of Ichneumonoidae in the eucalyptus stands with and without an understory, especially those of the families Braconidae and Ichneumonoidae, may indicate the presence of hosts such as *Oxydia vesulia* (Cramer, 1779) (Lepidoptera: Geometridae), a pest of *E*. *grandis* and of *Croton floribundus* Spreng (Euphorbiaceae) [67]. Chalcidoidea, belonging to this superfamily, is an extremely diverse group, with an estimated 500,000 species [68].

The Eulophidae, with similar numbers of individuals in the plantations with and without an understory, are important pupa parasitoids of the primary pest of eucalyptus, *Thyrinteina leucocerae* Rindge (Lepidoptera: Geometridae), parasitized by *Palmistichus elaeisis* Delvare and LaSalle, 1993 [69].

Differences in the number of individuals of the Hymenoptera parasitoids, with the higher frequency from the end of the rainy season in the region (June), may be due to the fact that the rainfall alters the foliar plant characteristics, and thus feeding and development of the herbivore, with consequences on the interactions at the higher trophic levels [70]. Plants in tropical forests with dry weather have leaves with short life which are thinner and high in nitrogen content [71] and with greater defenses against the herbivores than those in the rainforests [72,73]. This can increase the developmental stage of the caterpillars eating leaves with high defense and, thus exposing them to predators and parasitoids for longer periods [74,75,76]. Moreover, the smaller parasitoids are more affected by the dry conditions due to the increased drying [77].

## Conclusions

The presence of an understory within the eucalyptus plantations seemed to hinder the movement of some Hymenoptera species, although similar family richness was found in understory present and absent areas. This leads to lower diversity and higher dominance of Mymaridae over the remaining Hymenoptera families. Although some families may benefit from the higher plant diversity in understory present plantations, the fragments of native vegetation surrounding the plantations might override this benefit as it probably acts as a source of parasitoids that may better penetrate plantations without understory. The presence of parasitoids was evaluated but their effect in form of parasitism rates of pests and ultimately lowering damage to eucalyptus plantations still needs to be evaluated, but we showed the first insights on how the understory and the climatic season may change biological control prospects
